# Predictive value of vitamin D levels in neonatal respiratory failure: a retrospective study

**DOI:** 10.3389/fped.2025.1680809

**Published:** 2025-11-19

**Authors:** Chenlu Zhang

**Affiliations:** Department of Pediatircs, Tianjin Medical University Baodi Hospital, Tianjin, China

**Keywords:** neonatal, vitamin D deficiency, pregnant woman, perinatal, related factors

## Abstract

**Objective:**

To retrospectively analyze the predictive value of an early neonatal vitamin D (vit D) level for neonatal respiratory failure in newborns.

**Methods:**

The data of 220 hospitalized neonates, including 114 boys (51.8%) and 106 girls (48.2%), were collected from March 2021 to March 2023. Neonates were divided into respiratory failure (*n* = 52) and non-respiratory failure groups (*n* = 168) based on the respiratory status at the time of admission. Serum 25-hydroxy vitamin D [25(OH)D] levels were determined using a chemiluminescence immunoassay. The influence of serum 25(OH)D levels and perinatal factors on neonatal respiratory failure were analyzed. The predictive value of 25(OH)D for respiratory failure was evaluated.

**Results:**

Serum 25(OH)D (OR = 0.896, 95% CI = 0.817–0.984) and birth weight (OR = 0.999, 95% CI = 0.998–1.000) were independent protective factors against neonatal respiratory failure, while gestational age < 34 weeks (OR = 8.293, 95% CI = 1.326–51.863) was an independent risk factor for neonatal respiratory failure (*P* < 0.05). The area under the receiver operating characteristic curve (AUC) for 25(OH)D alone was 0.620 (95% CI = 0.531-0.708). However, combining the 25(OH)D level with gestational age and birth weight improved the AUC to 0.868 (95% CI = 0.812-0.925) with a sensitivity of 0.750 and 1-specificity of 0.845 (*P* < 0.001).

**Conclusion:**

25(OH)D deficiency may increase the risk of neonatal respiratory failure. Combining 25(OH)D with gestational age and birth weight was shown to have better predictive value for respiratory failure than serum 25(OH)D alone.

## 1 Introduction

Neonatal respiratory failure refers to the inability of newborns to maintain respiratory homeostasis, resulting in ventilation-perfusion mismatch and gas exchange dysfunction. It is a common neonatal emergency worldwide. Over the past seven decades, the fatality rate in high-income countries has decreased significantly; however, due to the lack of neonatal wards and neonatal intensive care units, the mortality rate of neonatal respiratory failure in low - and middle-income countries remains high (1).

Vitamin D (vit D) is a lipid-soluble pre-hormone steroid that has an important role in many physiologic activities. Vit D, upon binding to vit D receptor, interacts with activator and calcium-binding proteins, initiating genomic actions that regulate skeletal and calcium homeostasis, cell proliferation, differentiation, wound healing, repair mechanisms, immune modulation, and inflammatory responses (2). Vit D is expressed in fetal pulmonary cells (3), in which vit D participates in lung development, immune system maturation, and improving pulmonary function (4). Globally, vitamin D deficiency is more common among pregnant women and newborns than in the general population. There are significant differences in vitamin D status among different regions of the World Health Organization, with the highest prevalence of vitamin D deficiency among pregnant women and newborns in Southeast Asia (5). Serum 25-hydroxy vitamin D [25(OH)D] is the major circulating form of vit D and is therefore considered the most important indicator of vit D status worldwide (6).

In addition, vit D may modulate immune functions by enhancing monocyte and macrophage function in innate immunity via induction of cathelicidin, which is an antimicrobial peptide with properties that kill bacteria, viruses, and fungi. Vit D regulates T and B cells in adaptive immunity, especially by aiding the differentiation of T cells into regulatory T cells (Tregs), which are crucial for preventing autoimmune responses (7). Moreover, vit D modulates cytokine production by decreasing pro-inflammatory cytokines and increasing anti-inflammatory cytokines, thereby helping to control inflammatory responses. Many immune cells express vit D receptor and vit D binding to vit D receptor regulates the expression of genes responsible for the immune response. Therefore, vit D has been reported to be an immune modulator (7, 8).

Previous studies have paid more attention to the high correlation between vitamin D in mothers and newborns. The vit D level has been shown to influence postnatal respiratory outcomes. But few studies have focused on neonatal respiratory failure and the connection with vit D. Therefore, this study investigates the predictive value of the serum 25(OH)D level on neonatal respiratory failure was investigated based on the relationship to respiratory illnesses in early infancy.

## 2 Materials and methods

### 2.1 Study subjects

The following retrospective study analyzed the clinical data from 220 neonates ≤3 days old, who had been admitted to Tianjin Medical University Baodi Hospital between March 2021 and March 2023. The Ethics Committee of Tianjin Medical University Baodi Hospital (2021-E-0301) provided ethical clearance for this study. The study adhered to the Declaration of Helsinki. Guardians were informed and provided written informed consent for participation in the study.

The inclusion criteria were as follows: neonates ≤3 days old with complete clinical data; respiratory failure occurring within 24 h after production; maternal age between 18 and 45 y. The postnatal C-reactive protein (CRP) level was determined to be normal or abnormal based on neonatal sepsis diagnostic criteria, the postnatal procalcitonin (PCT) level, and the hourly adjusted PCT cut-off value after birth. Respiratory failure was diagnosed according to the 5th edition of the practical neonatology diagnostic criteria, as follows: 1. clinical indicators, including inspiratory depression, groan, central cyanosis, refractory apnea, decreased activity, and respiratory rate >60 beats/min; 2. laboratory indicators, including PACO_2_ > 60 mmHg, PO_2_ < 50 mmHg, or oxygen saturation <80% at an FiO_2_ = 100%; and c. arterial blood gas pH < 7.2. Clinical indicators plus one or more laboratory indicators facilitated the diagnosis of respiratory failure. The causes of respiratory failure include neonatal pneumonia and neonatal respiratory distress syndrome. Anti-infective and respiratory support treatments were administered after admission.

The exclusion criteria were as follows: women with triplets or more; pregnant women who had received vit D or vit A + D supplements for >2 weeks; women with a history of long-term use of drugs affecting vit D metabolism, such as antiepileptics, antineoplastics, antiretrovirals, antituberculosis medications, or glucocorticoids; women who smoked cigarettes or consumed alcohol during pregnancy; neonates >3 d old; and neonates with severe anemia, congenital malformations, severe asphyxia during the perinatal period, or suspected genetic/metabolic disorders.

### 2.2 Clinical materials

All medical records were collected, systematically re-evaluated, and data extracted based on the diagnostic criteria of the relevant literature. Neonatal baseline data upon admission included gender, gestational age, fetus number (Single or twin pregnancies), birth weight, percentile, and laboratory results [procalcitonin, CRP, and 25(OH)D] from blood samples obtained within 1 h. Birth seasons were included because seasonal factors have a significant impact on Vit D levels (9). Prenatal maternal data included general demographic data, pregnancy complications, and para-clinic test results.

### 2.3 Sample collection and testing

Because mothers who resided locally did not take a vit D supplement during pregnancy, the possibility of vit D deficiency in newborns was high. 25(OH) levels are routinely checked in newborns to determine the need for vit D supplementation. The Obstetrics Department in our hospital routinely delays umbilical cord ligation for 30 s after delivery to ensure the highest hemoglobin level possible in the newborn. Then, peripheral venous blood samples (3–4 mL) were collected from the femoral vein or radial artery of neonates within 1 h of admission under sterile conditions by specialist nurses with >15 years of experience. The peripheral venous blood samples were allowed to rest for 10–20 min, then centrifuged at 3,000 r/min for 15 min at 4 °C. The serum was separated and stored at −80 °C for subsequent analysis. Serum 25(OH)D was tested by CLIA using the iFlash 3,000G analyzer (YHLO, city, state, country). Tests were carried out using magnetic bead-acridinium ester technology with the 25(OH)D CLIA reagent kit (catalog number, 2021021; http://www.szyhlo.com/products_details/3.html).

### 2.4 Grouping criteria

The *Practical Diagnostic Criteria for Neonatology* (5th edition) was used as a reference. Clinical indicators are inspiratory depression, moaning, central cyanosis, refractory apnea, reduced activity, and respiratory rate >60 breaths/min. The laboratory indicators include PaCO_2_ > 60 mmHg, arterial blood gas (ABG) or FiO_2_ at 100% with a PO_2_ < 50 mmHg or SaO_2_ < 80%, and an ABG pH < 7.2 (10).

Subgroups were classified based on Vit D level: A 25(OH)D level of ≥20 ng/ml(50 nmol/L) was considered sufficient, 12–20 ng/mL(30–50 nmol/L) was insufficient, and <12 ng/ml(30 nmol/L) was deficient (9).

Subgroups were classified based on an abnormal CRP level, as follows (11): CRP ≥ 3 mg/L group within 6 h after birth; CRP ≥ 6 mg/L group from 6 to 24 h after birth; and CRP ≥ 10 mg/L within 24 h after birth.

Subgroups were classified based on abnormal PCT levels as follows (12): PCT level ≥0.5 ug/L group within 6 h after birth; PCT level ≥2 ug/L group between 6 and 24 or between 48 and 60 h after birth; PCT level ≥5 ug/L between 12 and 18 or 36 and 48 h after birth; PCT level ≥10 ug/L between 18 and 36 h; and PCT level ≥1 ug/L between 60 and 72 h.

### 2.5 Statistical methods

SAS 9.4 was used for data analysis. Categorical data are presented as counts and percentages. The Kolmogorov–Smirnov test was performed to check normality. Data that were normally distributed are expressed as the mean ± standard deviation (mean ± SD). Comparisons were made using an independent samples t-test. Non-normally distributed data are presented as the median and interquartile range [M (IQR)] and the Kruskal–Wallis test, a non-parametric rank-sum test, was used for analysis. ROC curves were used to demonstrate the predictive value of the newborn serum 25(OH)D concentration in conjunction with other markers for risk of respiratory failure. The Youden index, sensitivity, specificity, and AUC were calculated. A *P* < 0.05 was considered an indicator of statistical significance.

## 3 Results

### 3.1 comparison of neonatal baseline characteristics

Based on a comparative analysis, statistically significant differences were detected in the incidence of births in the winter (40.4% vs. 19.1%; *P* = 0.011), preterm births <34 weeks gestations (42.3% vs. 4.8%; *P* < 0.001), and a decreased mean birth weight (2,208.56 ± 660.98 g vs. 2,962.59 ± 697.36 g; *P* < 0.001) between the respiratory and non-respiratory failure groups. No statistical difference was detected in the gender distribution (male, 57.7% vs. 50.0%; *P* = 0.332), number of fetuses (singleton, 86.5% vs. 87.5%; *P* = 0.856), and birth weight percentile (small-for-gestational-age, 19.2% vs. 11.9%; *P* = 0.159; Table 1).

**Table 1:** Comparison of baseline characteristics among neonates from different groups.

Indices	Respiratory failure group *n* = 52(%)	Non-respiratory failure group *n* = 168(%)	*χ*^2^/t	*P*
Season of birth			11.178	0.011
Spring	12 (23.1)	57 (33.9)		
Summer	7 (13.4)	41 (24.4)		
Autumn	12 (23.1)	38 (22.6)		
Winter	21 (40.4)	32 (19.1)		
Gender			0.941	0.332
Male	30 (57.7)	84 (50.0)		
Female	22 (42.3)	84 (50.0)		
Fetus number			0.033	0.856
Singleton	45 (86.5)	147 (87.5)		
Twins	7 (13.5)	21 (12.5)		
Gestational age (weeks)			52.383	< 0.001
<34	22 (42.3)	8 (4.8)		
34–36^+6^	19 (36.5)	61 (36.3)		
≥37	11 (21.2)	99 (58.9)		
Birth weight (g)	2,208.56 ± 660.98	2,962.59 ± 697.36	−6.896	< 0.001
Birth weight percentile			3.673	0.159
Small for gestational age	10 (19.2)	20 (11.9)		
Appropriate for gestational age	39 (75.0)	125 (74.4)		
Large for gestational age	3(5.8)	23(13.7)		

### 3.2 Comparison of neonatal infection indices in different subgroups

CRP and PCT levels fluctuate greatly in the early stage of neonates. Therefore, in this study a value less than the cut-off value based on age characteristics was selected as normal and a value higher than the cut-off value was selected as the abnormal group. The grouping results are shown in Table 2. The incidence of respiratory failure in neonates with an abnormal PCT level was significantly higher than neonates without respiratory failure (*χ*2 = 21.638; *P* < 0.001), while a normal CRP level had no significant effect on the incidence of neonatal respiratory failure (*P* = 0.628), as shown in Table 2.

**Table 2:** Comparison of neonatal infection indicators in different groups.

Grouping	Respiratory failure group *n* = 52(%)	Non-respiratory failure group *n* = 168(%)	*χ^2^/F*	*P*
Procalcitonin			21.638	<0.001
Normal	36 (69.2)	157 (93.5)		
Abnormal	16 (30.8)	36 (21.5)		
CRP				0.628
Normal	48 (92.3)	155 (92.3)		
Abnormal	4 (7.7)	13 (7.7)		

CRP, C-reactive protein.

### 3.3 Comparison of maternal factors

When compared to the control group, more mothers in the respiratory failure group had residences in rural areas than the non-respiratory group (63.5% vs. 47.0%; *P* = 0.038). The incidence of cesarean delivery was significantly higher in the respiratory failure group than the non-respiratory failure group: (80.8% vs. 64.9%; *P* = 0.031. The percentage of mothers with insufficient or excessive weight gain was higher in the respiratory failure group than the non-respiratory failure group (28.8% vs. 13.1% and 42.4% vs. 50.0%, respectively; *P* = 0.029). Moreover, placental immaturity, especially grades 0–1, was noted more frequently in the respiratory failure group (53.9% vs. 31.6%; *P* = 0.004). Prenatal dexamethasone injection was more frequently performed in the respiratory failure group (48.1% vs. 20.2%; *P* = 0.000). Other maternal factors, including productive age of the pregnant women, education level, prenatal body mass index, and gestational hypertension, did not differ significantly between the two groups, which may indicate that these factors are less influential on neonatal respiratory failure outcomes (Table 3).

**Table 3:** Analysis on the effect of maternal factors on neonatal respiratory failure.

Indices	Respiratory failure group *n* = 52(%)	Non-respiratory failure group *n* = 168(%)	χ^2^/t/Z	*P*
Productive age of the pregnant women (years)			0.782	0.854
≤24	4 (7.7)	15 (8.9)		
25–29	19 (36.5)	53 (31.6)		
30–34	24 (46.2)	78 (46.4)		
≥35	5 (9.6)	22 (13.1)		
Residence			4.293	0.038
Urban cities	19 (36.5)	89 (53.0)		
Rural area	33 (63.5)	79 (63.5)		
Education				
Bachelor's degree or above	14 (26.9)	63 (37.5)	1.953	0.162
Vocational education or below	38 (73.1)	105 (62.5)		
Jobs			0.235	0.628
Housewife	24 (46.2)	84 (50.0)		
Non-housewife	28 (53.8)	84 (50.0)		
Parturition mode			4.657	0.031
Natural birth	10 (19.2)	59 (35.1)		
Caesarean	42 (80.8)	109 (64.9)		
Gestational diabetes mellitus			0.011	0.918
Yes	22 (42.3)	72 (43.1)		
No	30 (57.7)	96 (56.9)		
Prenatal BMI (kg/m^2^)			3.369	0.338
≤18.5	1 (1.9)	6 (3.6)		
18.6–23.9	23 (44.2)	77 (45.8)		
24–27.9	10 (19.2)	46 (27.4)		
≥ 28	18 (43.7)	39 (23.2)		
Weight gain during pregnancy			7.082	0.029
Insufficient	15 (28.8)	22 (13.1)		
Optimal	15 (28.8)	62 (36.9)		
Excessive	22 (42.4)	84 (50.0)		
Fetus number			3.191	0.074
Singleton	23 (44.2)	98 (58.3)		
Twins or multiple births	29 (55.8)	70 (41.7)		
Miscarriage history			0.657	0.418
Yes	17 (32.7)	65 (38.9)		
No	35 (67.3)	102 (61.1)		
Premature rupture of amniotic fluid			0.001	0.971
Yes	18 (34.6)	57 (34.3)		
No	34 (65.4)	111 (65.7)		
Gestational hypertension			2.508	0.113
Yes	16 (30.8)	34 (20.2)		
No	36 (69.2)	134 (79.8)		
Pre-eclampsia			0.433	0.511
Yes	8 (15.4)	20 (11.9)		
No	44 (84.6)	148 (88.1)		
Assisted reproductive technology				0.594
Yes	0 (0.0)	5 (6.0)		
No	52 (100.0)	163 (94.0)		
Placental abnormality			0.839	0.360
Yes	5 (9.6)	10 (6.5)		
No	47 (90.4)	158 (93.5)		
Placental maturity grade			11.062	0.004
0∼1	28 (53.9)	53 (31.6)		
II	23 (44.2)	97 (57.7)		
III	1 (1.9)	18 (10.7)		
Intramuscular injection of dexamethasone prenatally			15.680	<0.001
Yes	25(48.1)	34(20.2)		
No	27(51.9)	134(79.8)		

BMI, body mass index.

### 3.4 Comparison of neonatal serum vit D level

Table 4 compares the serum 25(OH)D levels between the respiratory and non-respiratory failure groups, showing several key statistical findings. The mean 25(OH)D level was significantly lower in the respiratory failure group than the non-respiratory failure group (15.11 ± 4.71 ng/mL vs. 16.80 ± 4.81 ng/mL; *t* = −2.215, *P* = 0.028). There was a significantly higher percentage of patients in the respiratory failure group than the non-respiratory failure group with 25(OH)D levels <12 ng/mL (9.45% vs. 30.55%, *χ*² = 5.521, *P* = 0.023). There was no significant difference in the 2 groups of patients with 25(OH)D levels ranging from 12–20 ng/mL (32.15% vs. 62.50%; *χ*² = 0.140, *P* = 0.710). Compared to the respiratory failure group, A significantly higher percentage of patients in the non-respiratory failure group had a 25(OH)D ≥20 ng/mL (22.60% vs. 10.40%; *χ*² = 3.047, *P* = 0.081).

**Table 4:** Comparison of 25(OH)D levels in neonates from different groups.

Parameter	Respiratory failure group *n* = 52(%)	Non-respiratory failure group *n* = 168(%)	t/χ^2^	*P*
25(OH)D (ng/mL)	15.11 ± 4.71	16.80 ± 4.81	−2.215	0.028
Distribution of 25(OH)D (ng/mL)			6.751	0.034
<12 ng/mL (%)	15 (28.9)	25 (14.9)	5.521	0.023
12–20 ng/mL (%)	31 (59.6)	105 (62.5)	0.14	0.710
≥20 ng/mL (%)	6 (11.5)	38 (22.6)	3.047	0.081

### 3.5 Binary logistic regression analysis

Table 5 shows the results of binary logistic regression analysis on maternal and neonatal factors associated with neonatal respiratory failure, including those factors with *p* values < 0.05. Neonates born before 34 completed weeks of gestation had a significantly higher risk of respiratory failure (OR = 8.293, 95% CI = 1.326–51.863; *P* = 0.024). Therefore, neonates born at <34 weeks gestation had an 8.3-fold increased risk of developing respiratory failure compared to neonates born after 37 completed weeks of gestation. Lower levels of 25(OH)D were associated with an increased probability of neonatal respiratory failure (OR = 0.896, 95% CI = 0.817–0.984; *P* = 0.021). The probability respiratory failure increased by 10% approximately for each 1 unit incremental decrease in the 25(OH)D concentration. Birth weight was also significantly associated with respiratory failure (OR = 0.999, 95% CI = 0.998–1.000; *P* = 0.014). Although small, this effect suggested that a lower birth weight was associated with a slightly increased risk for respiratory failure with each 1 g reduction in birth weight associated with a marginal increase in risk. Excessive maternal weight gain during pregnancy was not associated with neonatal respiratory failure (*P* = 0.086).

**Table 5:** Binary logistics regression analysis of neonatal respiratory failure.

Indicators	β	Waldχ^2^	*P*	OR	95% CI	
Birth seasons						
Born in winter		3.425	0.331			
Born in spring	−0.919	2.797	0.094	0.399	0.136	1.171
Born in summer	−0.744	1.447	0.229	0.475	0.141	1.598
Born in autumn	−0.846	2.026	0.155	0.429	0.134	1.376
Gestational age (weeks)						
Gestational age≥37 weeks		9.465	0.009			
<34	2.115	5.115	0.024	8.293	1.326	51.863
34–36^+6^	0.108	0.028	0.866	1.114	0.318	3.904
Birth weight (g)	−0.001	6.055	0.014	0.999	0.998	1.000
25(OH)D(ng/mL)	−0.109	5.291	0.021	0.896	0.817	0.984
Vaginal delivery	−0.620	1.541	0.215	0.538	0.202	1.432
Excessive weight gain during pregnancy		4.897	0.086			
Insufficient weight gain during pregnancy	0.560	1.093	0.296	1.751	0.613	5.001
Optimal weight gain during pregnancy	−0.742	2.171	0.141	0.476	0.177	1.278
Living in urban cities	−0.797	3.376	0.066	0.451	0.193	1.055
Intramuscular injection of dexamethasone prenatally	−0.164	0.093	0.760	0.849	0.296	2.432
Placental maturity grade III		0.583	0.747			
Placental maturity grade 0–I	0.028	0.001	0.982	1.028	0.096	10.955
Placental maturity grade II	0.380	0.115	0.735	1.462	0.162	13.157

25(OH)D: serum* 25-hydroxy vitamin D.

### 3.6 Combination of the serum 25(OH)D level and multiple indicators in diagnosing neonatal respiratory failure

**Figure 1:**
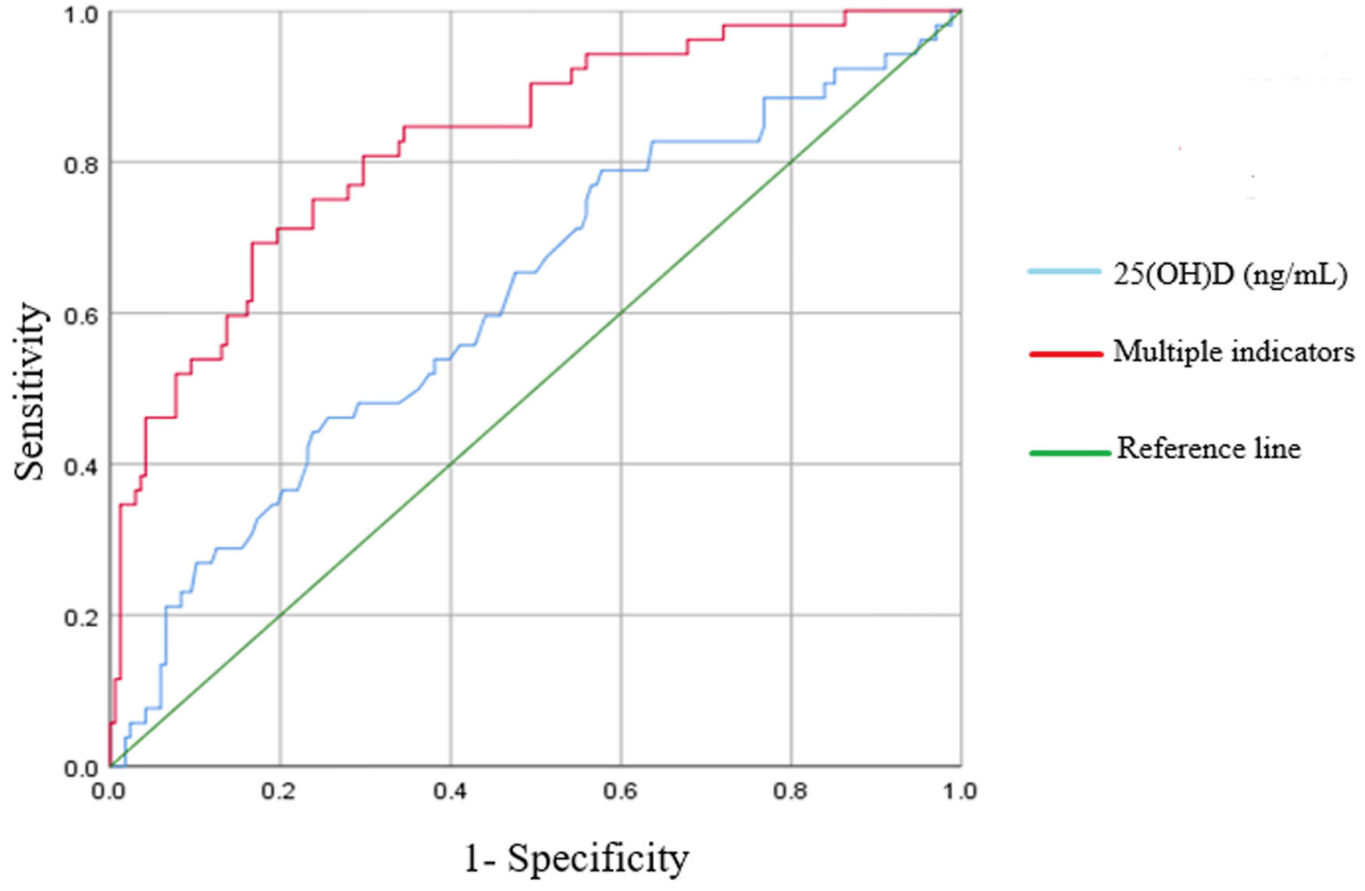
ROC curve comparison of 25(OH)D alone and 25(OH)D combined with multiple indicators in predicting the respiratory failure.

The receiver operating characteristic (ROC) curve for diagnosing neonatal respiratory failure based on the serum 25(OH)D concentration is shown in Figure 1. The area under the ROC curve (AUC) for serum 25(OH)D was 0.620 (95% CI = 0.531–0.708) with the largest Youden index of 0.211 when the cut-off value was 17.6 ng/mL. This finding suggests a sensitivity of 0.423 and a specificity of 0.788 at this cut-off value. The ROC curve remarkably improved in identifying respiratory failure by combining the serum 25(OH)D level with gestational age and birth weight. The AUC for multiple indicators reached 0.868 (95% CI = 0.812–0.925) with a maximum Youden index = 0.595. This combination yielded a sensitivity of 0.750 and specificity of 0.845, reflecting a higher predictive value for neonatal respiratory failure than single indicators (*P* < 0.001).

## 4 Discussion

Our study confirmed that preterm infants at a lower gestational age and birth weight have a higher predisposition to respiratory failure. Among preterm infants <34 weeks gestation, antenatal glucocorticoid exposure was associated with lower rates of acute respiratory distress syndrome but preterm infants still formed most neonatal respiratory failure cases. This finding suggests that close observation of neonatal vit D deficiency is required among neonates with a risk of respiratory complications.

Vit D has been associated with pulmonary function and inflammation. The effects of vit D on inflammatory cells, including dendritic cells, macrophages, T-cells, B-cells, and the structural epithelial cells, are important in mitigating pulmonary diseases (13, 14). Vit D modulates the immune system to enhance lung defense mechanisms against infections and inflammation, which are major contributors to respiratory failure (15). Furthermore, vit D supports pulmonary function by promoting the development of surfactant and improving alveolar fluid clearance, both of which help prevent pulmonary edema (16). Proper maturation of the respiratory system is all the more important in preterm infants because of the risk for respiratory complications due to immature lung development. There is a very close link between vit D levels and respiratory failure in preterm infants (17, 18), which is consistent with the results of the current study.

Other reports have also focused on neonatal and childhood respiratory outcomes as a function of vit D levels (19, 20). In one study, vit D deficiency among preterm neonates was associated with a higher incidence of respiratory distress syndrome, surfactant treatment, and non-invasive ventilation (19). In another study, infants in whom vit D levels were initially low still had a higher rate of respiratory and infectious morbidities, even though neonatal vit D levels improved with supplementation at standard doses, raising the possibility that personalized vit D dosing is required (20).

Severe vit D deficiency in umbilical cord blood has been associated with an increased risk of early birth and respiratory problems (21). Despite standard supplementation, many infants still had inadequate vit D, which is associated with a higher incidence of respiratory and allergic disorders (22). Currently, available studies suggest that vit D deficiency may increase the risk of respiratory failure through several mechanisms. Vit D is essential for fetal lung development and vit D deficiency impairs alveolar formation and surfactant production, leading to respiratory compromise (23). Additionally, vit D regulates immune responses and vit D deficiency can weaken the body's ability to fight infections, making neonates more susceptible to pulmonary infections (e.g., pneumonia), which can progress to respiratory failure (24). Vit D also helps control inflammation and vit D deficiency may exacerbate cytokine release, worsening lung damage in conditions like acute respiratory distress syndrome (ARDS) (25). Moreover, vit D maintains calcium homeostasis. Vit D deficiency leads to hypocalcemia, which eventually impairs respiratory muscle function and may contribute to respiratory failure (26). In conclusion, vitamin D deficiency in newborns increases the risk of neonatal respiratory failure by regulating the immune system and inflammatory responses, affecting the development of lung structure and lung ventilation.

Vit D deficiency in children has also been linked to the risk of developing recurrent respiratory infections (27) and is associated with an increased frequency of hospitalization for respiratory infections (28). Lykkedegn *et al*. concluded that there is still too little information to confirm or refute vit D in preventing or curing neonatal ARDS and bronchopulmonary dysplasia (BPD) (26) but significant animal and *in vitro* experiments support such biological roles in pulmonary maturation. Specifically, vit D roles in pulmonary maturation include promotion of alveolarization and maturation of type II alveolar epithelial cells, further adding to the hypothesis that vit D deficiency is a modifiable risk factor for ARDS and BPD (29).

BPD is a chronic respiratory disease that continues into childhood. BPD is defined by hypoxemia, pulmonary hypertension, wheezing, and an increased risk of asthma. The etiology of BPD is not well understood but includes immaturity of pulmonary structure and biochemistry, surfactant deficiency, low antioxidant and immune defenses, and injury from hyperoxia, hypoxia, or barotrauma caused by ventilatory support. These processes disrupt pulmonary epithelial cells, leading to edema, inflammation, and possible exacerbation by infections (30). Confirmation of the hypothesis through further research should include therapeutic studies on the usefulness of vit D in neonatal respiratory disorders.

Vit D-associated regulatory genes are expressed during pulmonary development in mice and humans. Vit D also downregulates disintegrin and metalloproteinase expression (31), which has a role in lung development and function (32). Sakurai *et al*. emphasized the critical role of vit D in perinatal lung maturation, including alveolar epithelial-mesenchymal interaction, regulation of alveolar fibroblast proliferation, and apoptosis (33). Additionally, vit D exerts beneficial effects on the respiratory system by maintaining integrity of the epithelial barrier and reducing lung injury (34). Vit D facilitates alveolar fluid transport by regulating epithelial sodium channel activity, thereby decreasing pulmonary edema (35). Lung barrier integrity depends on cohesive and tight junctions between epithelial and endothelial cells that form the air-blood barrier. Destruction of this barrier increases vascular permeability, promotes paracellular fluid movement into pulmonary spaces, and exacerbates lung dysfunction (36, 37).

### 4.1 Limitations of this study

The limitations of this study include the following issues. First, the retrospective design and failure to account for other factors that could influence neonatal respiratory failure, such as maternal nutrition and infection history. Second, the sample size was small from one single center, and the difference in sample size between the two groups was significant. This factor may lead to some bias in the results or statistical analysis. Third, although a correlation between maternal and neonatal vit D levels was observed, supplementation with vit D may have some protective effects. The results should be confirmed in large-scale prospective studies with larger sample sizes and assessment of multiple variable interactions. In fact, studying the interactive role of other micronutrients and prenatal factors associated with vit D may provide a fuller understanding of neonatal health.

## 5 Conclusion

The combination of neonatal vit D levels, gestational age, and birth weight provides a highly effective predictive model for respiratory failure incidence after birth. Respiratory failure is predicted by vit D deficiency or insufficiency, especially in preterm newborns with a gestational age < 34 weeks and low birth weight. By combining the above indicators, the risk prediction of neonatal respiratory failure may be improved, and effective respiratory support and comprehensive treatment can be actively adopted to reduce the impact of respiratory failure on newborns.

## Data Availability

The original contributions presented in the study are included in the article/Supplementary Material, further inquiries can be directed to the corresponding author.
